# Interprofessional follow-up for people at risk of type 2 diabetes in primary healthcare – a randomized controlled trial with embedded qualitative interviews

**DOI:** 10.1080/02813432.2024.2337071

**Published:** 2024-04-08

**Authors:** Marit Graue, Jannicke Igland, Bjørg Frøysland Oftedal, Anne Haugstvedt, Hilde Kristin Refvik Riise, Vibeke Zoffmann, Anne Karen Jenum, David Richards, Beate-Christin Hope Kolltveit

**Affiliations:** a Department of Health and Caring Sciences, Western Norway University of Applied Sciences, Bergen, Norway; b Department of Global Public Health and Primary Care, University of Bergen, Norway; c Department of Health Sciences, University of Stavanger, Stavanger, Norway; d Department of Internal Medicine, Haukeland University Hospital, Bergen, Norway; e Julie Marie Centre, Rigshospitalet, Research unit for Women`s and Children`s Health, Copenhagen, Denmark; f Institute of Public Health Copenhagen University, Copenhagen, Denmark; g General Practice Research Unit, Department of General Practice, Institute of Health and Society, University of Oslo, Oslo, Norway; h Institute for Health Research, College of Medicine and Health, University of Exeter, Exeter, United Kingdom; i Vossevangen medical center, Voss, Norway

**Keywords:** Type 2 diabetes, primary healthcare, randomized controlled trial, qualitative, self-management

## Abstract

**Objective:**

To examine the effects of an empowerment-based interprofessional lifestyle intervention program among people at risk of type 2 diabetes on knowledge, skills, and confidence in self-management, health, psychological well-being, and lifestyle characteristics, and to explore the participants’ perceptions of participating in the intervention.

**Design and methods:**

In line with the Medical Research Council complex interventions research methods framework, we conducted a randomized controlled trial with embedded qualitative interviews in primary healthcare clinics in Norway between 2019–2021. Of the patients at risk (The Finnish Diabetes Risk Score Calculator (FINDRISC) ≥15 or Body Mass Index (BMI) ≥30) 142 accepted the invitation, and 14 participants from the intervention group participated in individual interviews after the 12-month follow-up. Our primary outcome was the Patient Activation Measure (PAM-13). Secondary outcomes were EQ-5D-5L, EQ-VAS, WHO-Overall health, WHO-Overall QOL, weight, height, waist circumference, and regularity of physical activity. We used thematic analysis to analyse the qualitative data.

**Results:**

There was no clinically relevant differences of neither the primary nor the secondary endpoints between intervention and control group. As to the qualitative data, we identified two distinct features: ‘Meaningful perspectives on lifestyle changes’ and ‘Lifestyle change is not a linear process due to challenges faced along the way’ putting ownership of their choices in life into picture.

**Conclusion:**

The negative results of the RCT stand in contrast to the findings given by the participants voices, perceiving the intervention as a key eye opener placing their health challenges in perspective. How to interpret these seemingly conflicting findings of participants being seen, heard, and understood, helping them to take more conscious ownership of their choices in life, and at the same time demonstrating no improvements in symptoms or measures, is a dilemma that needs further exploration. We should be careful to implement interventions that do not demonstrate any effects on the quantitative outcomes.

## Introduction

Type 2 diabetes is a disease with a risk of serious long-term complications such as stroke, heart disease, kidney failure, and amputation, and is estimated to be the ninth leading cause of death worldwide [1]. However, type 2 diabetes is often asymptomatic in its early stages and can remain undiagnosed for several years. As a result, vascular complications often occur at the time of type 2 diabetes diagnosis [2]. Results from the Norwegian population-based HUNT4 survey (2017–2019) showed that 11.1% of those who had HbA1c levels ≥48 mmol/mol (diagnostic criteria for type 2 diabetes) in the study had not been previously diagnosed [3]. Participants with undiagnosed diabetes had a poorer cardiovascular risk profile and a more unfavorable risk factor profile (a higher body mass index, waist circumference, and systolic blood pressure) than participants with known or no diabetes. Thus, the seriousness of an elevated risk of diabetes underlines the urgent need for early intervention and efficient and targeted follow-up tailored to patients’ needs and preferences. Early identification and intervention are important to prevent or delay the development of the disease and associated co-morbidities [4]. Thus, it is important to design cost-effective lifestyle prevention programs to prevent the development of the disease and its complications [5,6]. Lifestyle behaviours such as diet and physical activity are key issues in the prevention of diabetes [7]. Recent research from the Diabetes REmission Clinical Trial (DiRECT) has shown that sustained remission of type 2 diabetes is achievable through weight loss and tailored behavioural support [8]. However, empowering people to make beneficial choices to maintain their health and well-being may be challenging. The motivation for self-care activities to maintain health and prevent or delay the development of diabetes at an early stage might best be maintained when people are supported by an interprofessional team [9]. The evidence on integrated care approaches in specialist healthcare is well understood; however, role construction and boundaries within primary healthcare teams remain challenging [10]. It has been shown that patients report better experiences of chronic care when they perceive that they have access to the expertise of a broader team of healthcare professionals in primary healthcare [11]. Nevertheless, policy and organizational context affect the ability of teams to collaborate. In a scoping review of facilitators of interprofessional collaboration in primary healthcare [12] the need for further exploration of this area has been highlighted. Thus, the aims of this study were 1) to examine the effects of an empowerment-based interprofessional lifestyle intervention program in primary healthcare among people at risk of type 2 diabetes on knowledge, skills, and confidence in self-management, health and psychological well-being, and lifestyle characteristics, and 2) to explore participants’ perceptions of participating in the intervention.

## Material and methods

We designed a randomized controlled trial with embedded qualitative interviews in line with the United Kingdom Medical Research Council (MRC) complex interventions research methods framework [13] current at the time of undertaking this study. This highly influential framework provides researchers with guidance on how to investigate and understand the effectiveness of interventions which are both complex in terms of the number of intervention components and the context in which the intervention is delivered. The framework recommends collecting and analysing a combination of numerical and narrative data to both ascertain the effect size of an intervention and understand its processes, and the way in which it is delivered and received. Further information can be obtained from ClinicalTrials.gov ID: NCT04076384, date of trial registration: 2019-08-26. A total of 12 physicians and five registered nurses carried out the interprofessional lifestyle intervention program.

### Study population and setting

Participants were recruited from four primary healthcare clinics in the Western and Eastern parts of Norway in 2019. In Norway, the healthcare services are organised with a primary care physician (PCP) scheme that is a list-based system which aims to make sure that everyone receives the healthcare services they need at the right time. The PCPs consider whether they also will engage other professions e.g. registered nurses, at the clinic. This list-based system ensures services with high accessibility and continuity for all residents. We identified eligible patients from a survey conducted among all patients, 20–80 years of age, scheduled for regular consultations from May to December 2019 (*n* = 1404) [14]. Exclusion criteria were severe cancer, end-stage renal disease, severe depression, bipolar disorder, schizophrenia, and recorded cognitive deficiency. All patients identified to be at risk of developing type 2 diabetes (Finnish Diabetes Risk Score Calculator (FINDRISC) ≥15 [15] or Body Mass Index (BMI) ≥30) were defined as eligible participants (*n* = 411). Those at risk of developing diabetes were later contacted by phone by a secretary at each primary care practice and asked if they would like to participate in the intervention study. We excluded people who had declared a lack of motivation to be contacted again for follow-up studies after the initial survey study; people diagnosed with type 1 or type 2 diabetes; those who did not write, speak, or understand Norwegian; and those who were not invited because of logistic or organizational problems or were unavailable due to severe medical or psychological health challenges.

#### Sample size calculation

We performed power analysis based on the primary outcome (Patient Activation Scale-13 items). Based on a previous study of the psychometric properties of this instrument [16] we expected a mean (standard deviation (SD)) score of approximately 52 (14). As such, using 7 point (0.5 SD) in the calculation is derived from previous research and not a decision based on previous intervention studies using this instrument. Hereby, we estimated the required sample size to be 64 participants in each group to detect a treatment effect of 7 points (0.5 SD) with 80% power and a two-sided significance level of 0.05. To account for possible dropouts, we anticipated to increase the sample size to 77 (20% increase) in each group.

#### Randomisation and allocation concealment

An independent person performed block randomization stratified by study site using the ralloc-command in Stata with block sizes varying from 2 to 10.

#### The intervention group

The intervention was conducted in the Western and Eastern parts of Norway between 2019-2021. In addition to care as usual (scheduled consultations with the PCP), we used an empowerment-based counselling method (the Guided Self-Determination (GSD) approach) and a collaborative standardized medical report to ensure that the treatment and follow-up program aligned with current clinical guidelines. As the list-based PCPs scheme in Norway allows for differences in which professions that are part of the regular team, the participants in this study were recruited from four primary healthcare clinics with ≥3 PCPs and ≥1 nurse(s). Both nurses and physicians were employed at the clinic on a regular basis and not appointed just for the purpose of conducting this trial. The participants received one initial consultation with the nurse, followed by further consultations at the three-, six-, and 12-month follow-ups (Supplementary Table 1) with the PCP and the nurse The first consultation was set up to gain insights into the patients’ thoughts and wishes about their health situation and what they might find difficult to maintain health and wellbeing. Nurses collected information on participants’ life situations and individual challenges and considered individual resources and motivation for self-care activities. This insight was used within the interprofessional team to ensure that the PCPs competence was utilised providing person-specific care and that the needs of each participant were met during the consultations. In addition, we developed a standardized medical report with all the key elements of the medical and clinical observations and treatment to be fulfilled during consultations ensuring that the intervention was conducted according to national clinical guidelines (Supplementary Table 2). We considered that also incorporating a structured report of the GSD principles into the standardized medical records prompted more robust and durable attention to these key elements when using GSD in consultations as part of the regular team responsibilities for the intervention group in this trial. Hereby, the nurses and PCPs could safeguard the communication and collaboration between them and make sure that everyone in the interprofessional team worked together as intended.

The GSD method is an empowerment-based counselling approach that applies reflection-sheets and communication skills (mirroring, active listening, and values clarification response) as a tool in the consultation with people that have a health issue or having a difficult life situation at the moment. GSD has the potential to uncover important lifestyle choices and the foundation for patient decisions through communication and reflection, advocating empowerment and patient involvement [17]. GSD might increase the motivation to change health behaviour and thus self-manage their life to enhance their physical or mental well-being. By applying GSD in the consultations with patients their challenges and goals are discussed together between the patients and nurses. Although there are some steps in the GSD method the patients and nurses continuously move back and forth through communication and reflection. Patients fill out reflection-sheets at home and bring them into the consultation with the nurse. The reflection-sheets will enhance reflection and an awareness to their health situation and prepare them for the meeting with the nurse. Fidelity was tested after every fifth nurse consultation using a prefixed reflection sheet in which the nurses expressed how they applied the communication skills (mirroring, active listening, and values clarification response) during the consultation.

The nurses participated in a systematic training program to learn to use the GSD approach in individual consultations [18]. The program consisted of two workshops of lectures, a reading list to ensure quality in the use of GSD, as well as training and support when learning to practice advanced communication skills in a workshop, and thereafter by exercises in the clinic, supervised by the last author (BCHK).

#### The control group

Participants in the control group received care as usual which in these four clinics consisted of scheduled consultations with the PCP only. They underwent initially one individual examinations and follow-ups from their PCP, but the physicians could consider what was suitable for each patient. The control group was not provided nurse consultations.

### Data collection

#### Socio-demographic data

We recorded the following sociodemographic variables: sex, age, educational level (primary school, secondary education (high school), university/college ≤4 years, university/college >4 years), living situation (living with others or living alone), and work situation (working full-time, working part-time, on benefits, retired, or other).

#### Questionnaire data

To examine the effect of the intervention, we used the Patient Activation Measure (PAM −13) as the primary outcome. The instrument assesses individual knowledge, skills, and confidence integral to managing one’s health and healthcare and comprises 13 items on a 1–4-point Likert scale. The instrument also specifies the level of patient engagement according to the four activation levels. The raw score (range 0-11) can be converted into a 0–100-point scale: level 1 (≤47.0) ‘not believing activation important,’ level 2 (47.1–55.1)’ a lack of knowledge and confidence to take action,’ level 3 (55.2–67.0) ‘beginning to take action,’ and level 4 (≥ 67.1) ‘taking action.’ The Norwegian version of the questionnaire has been validated and has acceptable psychometric properties [16].

We used the following secondary outcomes to assess health status: EQ-5D-5L, EQ-VAS, and WHO-Overall health, and the WHO-Overall QOL and WHO-5 to measure psychological well-being. The EQ-5D-5L is a standardized instrument with five items, from level 1 ‘no problems’ to level 5 ‘severe problems,’ measuring different dimensions of health: mobility, self-care, usual activities, pain/discomfort, and anxiety/depression [19]. A weighted summary score with values between 0 and 1 was calculated, as well as a visual analog scale (EQ-VAS), with values between 0 (worst health imaginable) and 100 (best health imaginable). The overall quality of life and health were determined using two questions in the WHOQOL-BREF [20]. The two overall items read as follows: ‘How would you rate your quality of life?’ (WHO- Overall QOL), and ‘How satisfied are you with your health?’ (WHO-Overall health), both rated on a five-point Likert scale. Psychological well-being was measured using the 5-item World Health Organization Well-Being Index (WHO-5) [21]. The instrument has adequate validity as an outcome measure in intervention studies and is a sensitive and specific screening tool for depression. It consists of five positively phrased items that rate how well each of the statements apply to the person in the past two weeks. Each of the five items was scored from 0 (none of the time) to 5 (all the time). The items were summed and then multiplied by four to convert it to a 0 (absence of well-being) to 100 (maximal well-being) scale score. According to the WHO classification system relating scores to the consequences of the disease experienced by the individual, a low score may have a substantial negative impact on diabetes control [22]. A medical secretary ensured that the participants in both control and intervention groups completed all self-reported questionnaires at baseline, three, six and 12 months.

#### Lifestyle related data

We collected information on the following secondary outcomes to assess lifestyle-related data: weight, height, waist circumference, and regularity of physical activity (never, less than once a week, once a week, 2-3 times per week, nearly every day). Two or more days a week with activity is used as a low-threshold border for activity among the participants. To ensure reliable data, the same person responsible for the follow-up of the patients at each primary care practice received training and performed measurements of waist circumference, weight, and height to calculate BMI for all participants that were included in the study.

#### Qualitative interviews

To explore participants’ experiences participating in the intervention, a medical secretary, to whom the patients were unknown, invited 14 participants with variations in age and gender from the intervention group from all four clinics (six male and eight female; mean age, 57 years; age range 28-78 years) to participate in individual telephone interviews after the intervention was completed. All invitees consented.

Remote interviews were required due to Covid-19 restrictions at primary healthcare clinics all over Norway during this period (between January 2021 and June 2021). The last author (BCHK) performed all telephone interviews. The interview guide was initially developed by BCHK, a diabetes specialist nurse with extensive experience in general practice. After the development phase, two of the other co-authors, who were experienced qualitative researchers (MG and BFO), provided input to finalize the guide. The interview guide included areas such as experiences with participation in the GSD program and how they experienced conversation with the nurse and physician (Supplementary Table 3). The interviews lasted from 30 to 40 min.

### Ethical considerations

We obtained ethical approval from the South-Eastern Norway Regional Committee for Medical and Health Research Ethics (2019/28/REK South-East A) and from the Norwegian Centre for Research Data (NSD) (ID:821994). Within the MRC framework it is underscored that qualitative data can provide valuable insights into why an intervention fails or has unexpected consequences [13]. Thus, the application to REK South-East A included data on quantitative measures as well qualitative interviews. In accordance with international guidelines, we used the CONSORT and CONSERVE-CONSORT Extension Guidelines for Reporting Trial Protocols and Completed Trials Modified Due the Covid-19 Pandemic and Other Extending Circumstances [23]. In addition, the report was guided by the Consolidated criteria for Reporting Qualitative research (COREQ) [24].

### Data analysis

#### Quantitative data

We analysed demographic variables (age, sex, educational level, living situation, and work situation) descriptively using mean values and standard deviations for continuous variables and frequencies and percentages for categorical variables. We estimated the effect of the intervention at the three follow-up time points using linear mixed models with random intercept for person, fixed effect for time, and fixed effects for the interaction between group allocation and time. By omitting the fixed effect for group allocation from the model we achieved an adjustment for baseline values of the outcome [25]. We report the regression coefficients for the interaction at three, six and 12 months with 95% confidence intervals which can be interpreted as the mean difference of the outcome between the intervention group and the control group, after adjustment for baseline differences. We defined study participation as data on at least one primary or secondary outcome, and by using linear mixed models, we could include participants who dropped out during follow-up until drop-out, under the assumption that missing follow-up measurements were missing at random (MAR). Thus, the few participants who dropped out after three and six months were included in the dataset and contributed to the estimation of the effect at the time points where they participated. All analyses were performed using SPSS® Statistics 28 (IBM Corp., Armonk, NY, USA), STATA SE 16.0 and MP 17.0 (StataCorp LLC, College Station, Texas, USA).

#### Qualitative data

The interviews were transcribed verbatim. MG, BCHK, and BFO were familiarized with all data by reading the interviews repeatedly. We analysed, categorized, and interpreted our results by applying the six steps of Braun and Clarke’s thematic analysis [26]. We performed inductively using open coding and coded the transcripts separately. We then compared our codes to increase their trustworthiness and searched for patterns and themes in the data. We reviewed our themes by reading the interviews again, and developed preliminary themes, paying attention to our interview guide. We then decided on the final theme. Moreover, we invited the full group of authors to review the themes and provide inputs for the interpretation of the data. All authors reviewed the qualitative and quantitative data together to comment on the interpretation of the full data set, as well as implications for clinical practice.

## Results

### Quantitative data

**Figure 1.**
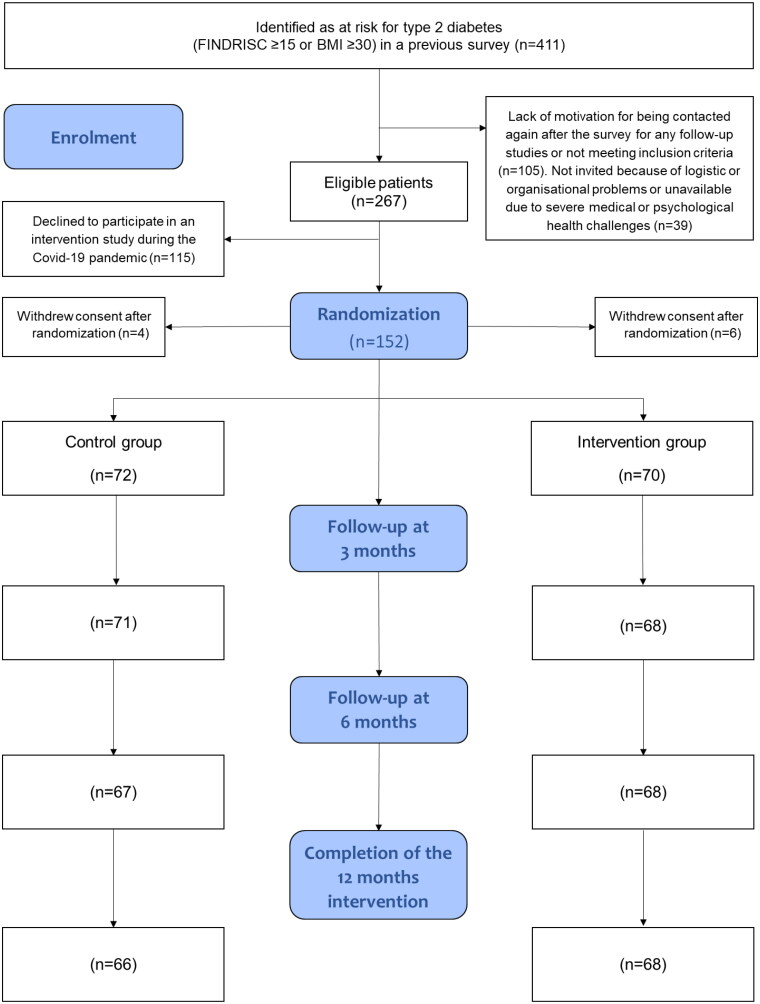
Flow of eligible patients invited to participate in the randomised trial among people risk of developing type 2 diabetes.

Figure 1 shows the total flow of participants in both groups throughout the trial. Of the total population of 411 adults who fulfilled the baseline criteria for participating in the trial, 144 patients were not eligible due to lack of motivation for being contacted again for follow-up studies after the initial survey study, not meeting the inclusion criteria, not invited because of logistic or organizational problems, or unavailable due to severe medical or psychological health challenges. In addition to other reasons, considering that this trial was conducted during the Covid-19 pandemic, 115 patients of the eligible patients declined to participate in the 12-months follow-up intervention. Furthermore, six participants in the intervention group and four in the control group withdrew their consent after randomization, and six participants were lost to follow-up in the control group and two in the intervention group. Table 1 presents the baseline characteristics of the 142 participants (intervention/control group, 70/72 participants): mean age, 57.9 years; age range 28-78 years, male/female 38%/62%. Overall, the randomization of the groups was well-balanced at baseline.

**Table 1.** Baseline characteristics of the study population.

	Total study population *N* = 142	Intervention *N* = 70	Control *N* = 72
Gender, *n* (%)			
Male	54 (38.0)	27 (38.6)	27 (37.5)
Female	88 (62.0)	43 (61.4)	45 (62.5)
Age, mean (SD)	57.9 (13.5)	59.4 (13.6)	56.5 (13.4)
Living situation, *n* (%)			
Living alone	31 (22.0)	16 (22.9)	15 (21.1)
Living with others	110 (78.0)	54 (77.1)	56 (78.9)
Educational level, *n* (%)			
Primary school	36 (25.4)	25 (35.7)	11 (15.3)
Secondary (high school)	69 (48.6)	27 (38.6)	42 (58.3)
University/College ≤4 years	31 (21.8)	15 (21.4)	16 (22.2)
University/College >4 years	6 (4.2)	3 (4.3)	3 (4.2)
Work situation, *n* (%)			
Full-time work	48 (33.8)	25 (35.7)	23 (31.9)
Part-time work	20 (14.1)	9 (12.9)	11 (15.3)
On benefits	16 (11.3)	8 (11.4)	8 (11.1)
Retired	49 (34.5)	25 (35.7)	24 (33.3)
Other	9 (6.3)	3 (4.3)	6 (8.3)
FINDRISC ≥ 15, *n* (%)	56 (39.4)	31 (44.3)	25 (34.7)
BMI ≥ 30	97 (71.9)	48 (70.6)	49 (73.1)
BMI, mean (SD)	32.3 (5.2)	32.4 (5.6)	32.2 (4.7)
LDL cholesterol (mmol/l), mean (SD)	3.4 (1.1)	3.4 (1.1)	3.5 (1.1)
Regularity of physical activity			
Never	4 (2.9)	1 (1.5)	3 (4.2)
Less than once a week	21 (15.0)	12 (17.7)	9 (12.5)
Once a week	23 (16.4)	13 (19.1)	10 (13.9)
2-3 times per week	53 (37.9)	22 (32.4)	31 (43.1)
Nearly every day	39 (27.9)	20 (29.4)	19 (26.4)

Abbreviation: SD: standard deviation; FINDRISC (The Finnish Diabetes Risk Score Calculator), BMI: body mass index), LDL (low-density lipoprotein).

**Figure 2.**
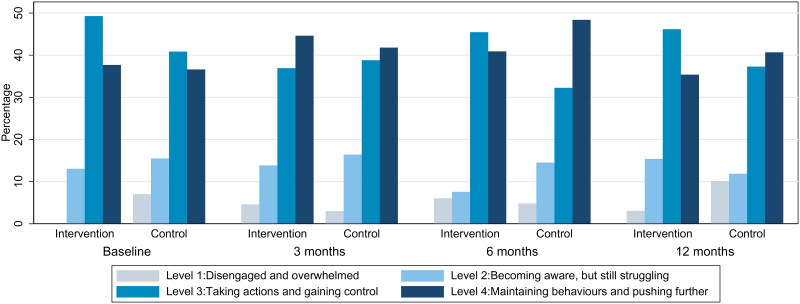
Distribution of PAM-levels in intervention group and control group at baseline and during follow-up.

**Figure 3.**
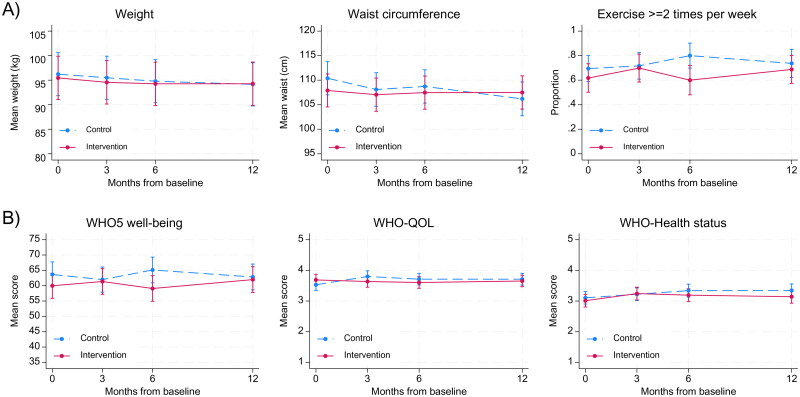
Mean and 95% confidence intervals for secondary outcomes in control group and intervention group at baseline and during follow-up.

We found no clinically relevant differences of between-group differences in our primary outcome (PAM-13). The differences between the treatment groups were small and non-significant (Table 2 and Figure 2). Moreover, PAM scores were relatively stable from baseline to the end of the study in both groups. The mean PAM score (range 0–100) declined from 69.6 at baseline to 67.3 at 12 months follow-up in the intervention group, while it increased from 66.7 to 68.2 in the control group. Also, at 12 months follow-up the number of people with PAM level 4 decreased from 26 to 23 in the intervention group and from 26 to 24 in the control group. Likewise, we found no clinically relevant differences of neither the secondary endpoint questionnaires between intervention and control group (EQ-5D-5L and WHO-5) (Table 3, Figure 3). A significant but small between-group difference was found for quality of life (WHO-QOL), indicating a slightly declining score in the intervention group, but not in the control group (Table 3). In addition, the between-group difference in waist circumference significantly increased at 12 months (Table 3) driven by a reduction in the control group and stable waist circumference in the intervention group (Table 3 and Figure 3).

**Table 2.** Effect of the Guided self-determination intervention on the patient activation (PAM) score and PAM-level 4 among persons with elevated risk of type 2 diabetes (*n* = 142).

		Intervention *N* = 70	Control *N* = 72	Between group difference B (95% CI)*	*p*-value
PAM score, mean (SD)					
Baseline		69.6 (12.3)	66.7 (12.8)		
3 months		69.2 (14.9)	68.7 (13.0)	−1.5 (−5.2, 2.3)	0.44
6 months		68.5 (13.5)	69.3 (14.2)	−2.1 (−5.9, 1.7)	0.28
12 months		67.3 (13.5)	68.2 (14.6)	−1.9 (−5.8, 2.0)	0.33
PAM level 4, *n* (%)					
Baseline		26 (37.7)	26 (36.6)		
3 months		29 (44.6)	28 (41.8)	2.8 (−14.1, 19.8)	0.74
6 months		27 (40.9)	30 (48.4)	−7.4 (−24.7, 9.8)	0.40
12 months		23 (35.4)	24 (40.7)	−5.3 (−22.4, 11.9)	0.55

* Difference in mean PAM score and percentage with PAM level 4 between intervention group and control group after adjustment for baseline value of outcome. Control group as reference group.

**Table 3.** Effect of GSD-intervention on secondary outcomes among persons in primary care with elevated risk of type 2 diabetes (*n* = 142).

	Baseline values, mean (SD)*		Between-groups difference 3 months		Between-groups difference 6 months		Between-groups difference 12 months	
	Intervention *N* = 70	Control *N* = 72	B (95% CI)**	*p*-value	B (95% CI)**	*p*-value	B (95% CI)**	*p*-value
Weight (kg)	95.5 (19.8)	95.6 (16.7)	−0.1 (−1.9, 1.6)	0.87	0.3 (−1.5, 2.1)	0.78	0.8 (−1.1, 2.6)	0.42
Waist circumference (cm)	107.9 (14.0)	111.4 (15.9)	1.0 (−1.6, 3.7)	0.43	0.8 (−1.7, 3.4)	0.52	**3.4 (0.7, 6.1)**	**0.01**
WHO-5	59.5 (15.9)	63.0 (17.7)	1.8 (−2.6, 6.2)	0.43	−3.6 (−9.1, 0.9)	0.12	1.6 (−3.0, 6.1)	0.50
WHO-QOL	3.7 (0.9)	3.5 (0.8)	**−0.3 (**−**0.5, −0.1)**	**0.01**	**−0.2 (**−**0.4, −0.0)**	**0.045**	−0.2 (−0.4, 0.0)	0.13
EQ5D	0.80 (0.16)	0.86 (0.11)	0.01 (−0.03, 0.05)	0.67	−0.03 (−0.06, 0.01)	0.19	−0.01 (−0.05, 0.03)	0.54
EQ5D-VAS	62.4 (19.9)	65.1 (17.1)	−1.4 (−6.1, 3.2)	0.55	−1.8 (−6.6, 2.9)	0.45	−4.5 (−9.4, 0.3)	0.07
WHO-Health	3.0 (0.9)	3.1 (0.9)	0.1 (−0.1, 0.3)	0.47	−0.1 (−0.3, 0.1)	0.46	−0.1 (−0.4, 0.1)	0.24
Exercise ≥ 2 times per week	42 (61.8)	50 (69.4)	−0.0 (−0.2, 0.1)	0.82	−0.2 (−0.4, −0.0)	0.01	−0.0(−0.2, 0.1)	0.55

* All values reported as mean and standard deviation except for exercise, which is reported as n(%).

** Difference in means, except for exercise, which is difference in proportion with exercise ≥ 2 times per week.

### Qualitative data

The patterns and themes were dominated by two distinct features in the data: *‘Meaningful perspectives on lifestyle changes’* and *‘Lifestyle change is not a linear process due to challenges faced along the way’*.

#### Meaningful perspectives on lifestyle changes

The participants expressed that conversation and relationship with the nurse based on the GSD approach became a key opener for seeing their health from a new perspective. Furthermore, they found that nurses focused more on establishing healthy habits to prevent type 2 diabetes rather than primarily losing weight. This new perspective came forward from one of them in the following manner.

*‘I once thought that I would never go on a diet. I have a new perspective on dietary advice now. Now it is all about my health and that I can be much healthier by changing my diet.’ (Participant 2)*

They perceived that the focus in the consultations shifted from primarily clinical or medical concerns towards their own perspectives on living their life within different circumstances, their individual health challenges, and thoughts on how to eventually make changes that would be attainable in their lives, and an atmosphere of trust was established in the consultation. The participants experienced that this form of conversation promoted motivation because of nurses’ insights and understanding. One of them said:

*‘I always experienced that the nurse believed in me. The nurse’s opinion was that it was my daily life I had to concentrate on, and the nurse saw what would be challenging for me and what worked for me. That insight is important for achievement’ (Participant 8)*

Some expressed that consultations with the nurse encouraged and empowered them to be able to address topics that were important to them in their present life situation, not only from a health and disease perspective. They said that being seen as a whole person and discussing with the nurse their challenges in life, possible strategies to solve the challenges, and, in the end, act upon these strategies, enhanced self-management. This was conveyed from one of them like this:

*‘The nurse got me not to focus on weight, but instead to think about what I ate. She got me to focus more on myself. She shined a spotlight on the totality of my life physically, psychologically, and socially. She gave me tips and advice about taking care of myself, how to plan and how to say ‘no’ sometimes which helped me take care of myself.’ (Participant 4)*

The participants noted that they experienced being seen as individuals who struggled with health challenges and that the nurse attentively had time to discuss these with them. They reported that the nurse focused on activating them in making goals for themselves and acting upon these goals. However, despite having motivation and support from nurses, they clearly expressed how difficult it was to stay motivated for an extended period. This leads us to the next theme.

#### Lifestyle change is not a linear process due to challenges faced along the way

The participants expressed that modifying lifestyle behaviours is not a linear process; thus, the support needs to be focused on their specific life situations and individual circumstances. They highlighted that they might have a desire to find a more favourable strategy to clear up their health challenges, but being motivated did not necessarily mean that they would succeed in their choices to engage in a healthier way of living. One of them expressed it as this:

*‘There has been changes in my diet…. My problem is that I have the desire to eat healthier and to exercise., there has been less of that. You must have a strong will to manage it.’ (Participant 2)*

They also conveyed that committing to their action plan when they faced challenges on their way was difficult. It was seen as particularly challenging if they had made several goals and should act upon all these goals at the same time. They tried to make it work, fully aware that nobody else could do the job for them; still, the process required support from others all the way. Moreover, shifting environments, such as weather circumstances and season altered the way they coped with lifestyle habits they had decided to change. One of them gave an example of the challenges faced:

*‘I try to continue as I did, but I must be honest and admit that I haven’t been so active in the winter as I was in the summer. It has to do with the fact that the weather is not so good, and it is snowing and cold outside.’ (Participant 1)*

Another participant conveyed the same view commenting on disappointment in failing to maintain the agreed upon new action plan. Although motivated, progress in losing weight was not realized due to general societal challenges and, notably, restrictions due to the Covid-19 pandemic. Participants experienced a change of focus in the consultations with the nurse towards an enhanced motivation for lifestyle changes; however, they also expressed how difficult it could be to be responsible for doing this change by themselves, while facing the challenge of Covid-19 when trying to do some changes. This was a hassle when fitness gyms and swimming pools were closed. One of them put it in this manner.

*‘It was difficult to be conscious of it and put it into practice. We spoke a lot about exercise and diet. The nurse tried to motivate me. But it was during Covid-19 and I didn’t get started as I thought I would.’ (Participant 14)*

Some also talked about other crises that they were struggling with. Their focus was on issues other than changing their lifestyle and ways of handling potential health risks, such as obesity. The difficulties of changing lifestyle habits and coping with other challenges they faced were expressed as follows:

*‘My situation in life was that I was focused on the crisis I was handling concerning my life situation. I had a slightly different focus, so I focused on that during the conversations with the nurse….’ (Participant 6)*

## Discussion

In our study we found no clinically relevant intervention effect on the primary outcome (patient activation), nor the secondary patient-reported outcomes, BMI, weight, waist circumference or exercise ≥ 2 times per week between intervention and control group. The scale scores for patient activation were high at baseline and stable throughout the 12-months intervention. In contrast, our qualitative results showed that the participants expressed high acceptability and satisfaction with this follow-up intervention conducted over a 12-month period. They perceived the intervention as a key eye opener, bringing a new perspective on their health challenges and helping them to take more conscious ownership of their choices in life. The predominantly positive interpretation of the participants voices in the qualitative interview results section, stands in contrast to the lack of intervention effect on primary and secondary outcomes in our RCT. These seemingly conflicting results warrant essential reflections.

The empowerment-based counselling method was perceived as meaningful because the shift from primarily clinical or medical concerns towards the patient’s own perspectives, promoting goal setting, and self-management engagement, was reported as useful by participants. They were gaining insights and understanding of the complexity of their health challenges in relation to their settings and life situations. Notably, some of them had not previously recognized or had ignored the fact that they were at risk of any substantial health problems. This finding is in line with the results of a study from the US [27] that emphasized that patients with prediabetes displayed less understanding of the ‘disease’ and perceived it to be less ‘chronic’ and ‘serious’ compared to people with manifest type 2 diabetes. One could speculate that the lack of a significant effect of the GSD intervention in our population might be related to the fact that people at risk for type 2 diabetes need time to realize that they are at risk of developing the condition. Interestingly, the intervention group reported a slightly lower quality of life than the control group. This makes us reflect upon quality of life as an expression of becoming aware of the seriousness of being diagnosed with type 2 diabetes. It can be problematic to be identified as an individual at risk for a chronic disease, however an early risk identification might enhance quality and length of life by early intervention. In line with this, it might also be of some ethical concern not to offer guidance in the healthcare system. Previously, it has been found that individuals with type 2 diabetes and macrovascular complications report lower health-related quality of life compared to the general population [28]. Given that type 2 diabetes is a serious concern that will not lessen without an effort to take on healthier behaviours, an important area of focus for supportive management is to address the psychological and emotional aspects of obesity-related conditions to support people’s awareness of the seriousness of risk factors [29]. Still, based on the lack of significant and substantial intervention effects in the current intervention it is difficult to interpret the seemingly conflicting findings that unquestionably hamper the implementation of interventions that may be positively valued by patients because they are seen, heard and understood when this knowledge is not followed by any distinct effects on clinical outcomes.

Another consideration on the lack of intervention effects is that this study was conducted in a ‘waiting room’ population of people scheduled for routine consultations with their PCP. It takes time to reach and achieve a pronounced awareness of individual health problems. Thus, although there is a lack of pronounced and immediate effects, counselling for more healthy behaviours at an early stage might still be important. Investing in type 2 diabetes prevention is a cost-effective strategy for at-risk individuals [6], since complications and costs for the healthcare system occur many years after diabetes onset. In our study, we sought to balance the need of patients and intensity of the intervention with organizational challenges in primary healthcare clinics. Nonetheless, it might be that the interval between the six- and 12-months consultations was too long. This was further complicated by conducting the intervention during the Covid-19 pandemic with locked-down primary healthcare clinics, as well as locked-down fitness gyms and very few supportive social gatherings. Still, it must be put into the picture that the economic and workload costs for the health personnel involved would require significant impacts for patients’ health to warrant the implementation in routine practice. This is in contrast to other more successful interventions to promote weight reduction, such as the DIRECT study where the success of intensified follow-up is clear [30].

The challenges of advocating an empowerment-based approach in practice are well known [31]. Thus, the transition from traditional consultation forms to the GSD approach has been perceived as demanding in previous research [32]. In a recent integrative review on the impact of GSD across different healthcare settings and diagnoses, it was shown that the counselling approach, although useful and well-accepted, continuous training and supervision of professionals are prerequisites for successful adoption in clinical practice [33]. Although the method is generally perceived to be helpful in primary healthcare consultations, previous research has shown that advanced communication skills require formal training and frequent repetition to reach the acquired competence among nurses in primary healthcare clinics [34]. Although the adoption of the interprofessional care model in this study fuelled a redefinition of roles and relationships that seemed to be positive for the healthcare professionals, organizational factors should be considered before putting the model into routine practice. This corresponds to the findings of Smeets et al. [35] highlighting that contextual factors, such as time constraints, influence team commitment and the success of a program. As the current organization in the Norwegian scheme of primary care clinics of small units with autonomous PCPs responsible for their own activities it might be a need for more comprehensive financial strategies to implement a different utilization of resources and competencies into a well-established health service. According to Blane et al. [36] primary healthcare clinics need more secure, sustained funding to engage in weight management, similar to well-resourced smoking cessation interventions. This matter is further illuminated in a study gaining a deeper understanding of the experiences of overweight patients in consultations with their primary healthcare physicians in Norway requesting them to take a more active role in talking about health challenges and risks related to poor lifestyle choices [37]. Thus, we question whether the implementation of the current intervention is feasible in the current situation with a trend of a rising workload in primary healthcare in Norway as well as in several other countries [38,39]. It has been shown that only half of the working time in general practice is used for face-to-face consultations [40]. Thus, the time available poses limited possibilities for identifying, monitoring, and supporting people at risk of developing future health problems without a more comprehensive financial strategy, enhancing team commitment and confidence in implementing empowerment-based, person-centered care in primary healthcare clinics. The potential to optimize primary care capacity by interprofessional collaboration between PCPs and nurses in primary care settings needs further exploration.

### Strength and limitations

Considering the lack of effects of the GSD intervention, the sensitivity and responsiveness of the primary outcome instrument may be questioned. Although, it might be considered a strength that the study used standardized, validated questionnaires, comparable to other studies, our study showed ceiling effects on most items for the PAM-13 instrument, which may reduce the sensitivity for identifying effects. As a rule of thumb, a 5–10% change of the instrument range score is considered meaningful for interpreting a difference when using patient-reported outcome measures [41]. It was a strength that we used a counselling approach that has a sound theoretical underpinning [18,42]. If delivered with sufficient fidelity, it has been used with success in a range of settings and diagnoses [33]. Moreover, it is considered a strength that we invited all individuals present in the waiting room area, fulfilling the inclusion criteria to participate in the study. Thus, the participants in the intervention group were reasonably representative of the people seeing their PCP for consultations. Although other aspects also might explain the lack of effects, it is beyond doubt, an obvious limitation to identify intervention effects that the trial was conducted during the Covid-19 pandemic. It would also have strengthened the study if we had reliable HbA1c measures available for all patients in the initial survey study. In addition, it might be considered ‘a minor intervention’ that the control group completed the same questionnaires at the same time intervals (0, 3, 6 and 12-months) as the intervention group, and as such became aware of their health risks. In addition, it might have influenced the actions from the PCPs that they had the medical responsibility for both the intervention and control group. After the first individual examinations consultation for all patients the intervention group underwent three consultations. It is unfortunate that we did not log how many consultations the PCP considered as suitable for each of the patients in the control group. An eventually negative development of the health of the participants in the control group might have influenced the follow-up. As part of the MRC framework, it is a strength that qualitative interviews can provide valuable insights into why an intervention fails or has unexpected consequences. As such, these findings shed some more lights to how the intervention was experienced by the patients in this context. We included both sexes and variation in age to get richer and more varied insights into their experiences during the intervention. Still, as in all intervention studies, it might be an option that the data may reflect some aspect of ‘eager to please’ because the intervention was conducted in the clinics where the patients were, and probably will be treated within the Norwegian list-based PCP scheme. Finally, performing the study in only four clinics, imply that one should be careful to transfer the findings from this context to other contexts.

## Conclusion

The negative results of the RCT stand in contrast to the findings given by the participants voices, perceiving the intervention as a key eye opener for seeing their health challenges in a new perspective. How to interpret these seemingly conflicting findings of participants being seen, heard, and understood, helping them to take more conscious ownership of their choices in life, and at the same time demonstrating no improvements in symptoms or measures, is a dilemma that needs further exploration. We should be careful to implement interventions that do not demonstrate any effects on the quantitative outcomes.

## Supplementary Material

Supplemental Material
