# Supplementary material for: Interprofessional follow-up for people at risk of type 2 diabetes in primary healthcare – a randomized controlled trial with embedded qualitative interviews
Source: Scand J Prim Health Care. 2024 Apr 8;42(3):450–62. doi: 10.1080/02813432.2024.2337071 (PMC11332301; doi:10.1080/02813432.2024.2337071)
Supplement: Supplemental Material [file IPRI_A_2337071_SM6131.docx]

**Supplementary Table 1.** Overview of the GSD program consisting of three follow-up consultations over 12-months.

**Supplementary Table 2.** Components available in medical records to ensure that medical aspects were monitored, and fidelity maintained in applying the GSD principles.

| Standardised components available in medical records to ensure that the following medical aspects were monitored to satisfy requirements in clinical guidelines.   - Review of the medications used by the patient. - Review of possible side effects of medications. - Review of the blood tests. - Blood pressure. - Height, weight, waist measurement. - Review of the patient`s physical activity in daily life. - Review of the patient`s dietary habits. - Review of the cardiovascular risk factors. |
| --- |
| Structured GSD principles available in medical records to ensure fidelity to the program.   - Today, what do you find difficult being at risk of type 2 diabetes? - Room for the condition in your life. - Your plans for changing your way of life. - Clarification of challenges in your life with an increased risk of developing type 2 diabetes. - Goals and intentions. - Your thoughts and feelings. - Your actions. - New strategies and plans. |

**Supplementary Table 3.** Interview guide.

| **Overall themes** |
| --- |
| What is your experience with participating in this study? |
| How did you experience the meetings with the nurse and the primary care physician regarding your health challenges? |
| What about the follow-up when participating in the study? Any changes? |
| Did you experience something negative? Did you experience something positive? |
| How did you experience the counselling from the nurse or the doctor? Is there anything that could have been different? |
| What is your experience with interprofessional follow-up? |
| Were there any changes in your health challenges during the last year? |
| Have you experienced changes in self-management during this time? |
